# Complement factor H regulates retinal development and its absence may establish a footprint for age related macular degeneration

**DOI:** 10.1038/s41598-018-37673-6

**Published:** 2019-01-31

**Authors:** Chrishne Sivapathasuntharam, Matthew John Hayes, Harpreet Shinhmar, Jaimie Hoh Kam, Sobha Sivaprasad, Glen Jeffery

**Affiliations:** University College London, Institute of Ophthalmology, London, EC1V9EL UK

## Abstract

Age related macular degeneration (AMD) is the most common blinding disease in those over 60 years. In 50% of cases it is associated with polymorphisms of complement factor H (FH), implicating immune vulnerability. But such individuals may exhibit abnormal outer retinal blood flow decades before disease initiation, suggesting an early disease footprint. FH is expressed in the retinal pigmented epithelium (RPE). During development the RPE is adjacent to the site of retinal mitosis and complex regulatory interactions occur between the relatively mature RPE and retinal neuronal precursors that control the cell cycle. Here we ask if the absence of FH from the RPE influences retinal development using a mouse CFH knockout (*Cfh*^−/−^) with an aged retinal degenerative phenotype. We reveal that from birth, these mice have significantly disrupted and delayed retinal development. However, once development is complete, their retinae appear relatively normal, although many photoreceptor and RPE mitochondria are abnormally large, suggesting dysfunction consistent with premature ATP decline in *Cfh*^−/−^. Total retinal mtDNA is also reduced and these deficits are associated shortly after with reduced retinal function. *Cfh*^−/+^ mice also show significant abnormal patterns of cell production but not as great as in *Cfh*^−/−^. These results reveal that not only is FH an important player in sculpting retinal development but also that the developmental abnormality in *Cfh*^−/−^ likely establishes critical vulnerability for later aged retinal degeneration.

## Introduction

Age related macular degeneration (AMD) is a major cause of blindness. The pathological changes are mainly located in the outer retina/choroid complex that consist of the photoreceptors, the retinal pigmented epithelium (RPE), Bruch’s membrane (BM) and the choroidal circulation. Here, inflammatory deposits appear, compromising blood flow to photoreceptors that have the greatest energy demand in the body and high mitochondrial density^1–4^. High metabolic demand is associated with rapid ageing^5,6^ where mitochondria also play a significant role. Mitochondrial decline is likely partly due to reduced perfusion resulting from BM deposition, restricting the oxygen required for adenosine triphosphate (ATP) production that is critical for cellular function. Declining mitochondrial integrity also drives inflammation and can signal cell death^7^. Hence, even in normal ageing, there is a 30% loss of central rod photoreceptors in human and rodent^8,9^, marking the retina as particularly vulnerable. In AMD there is an additional vulnerability, as 50% of patients have polymorphisms of complement factor H (FH)^10–12^, which is produced in the liver and RPE^13^. FH regulates inflammation. Further, there is a change of FH function that leads to an increase in C3b retinal deposition^14,15^. Hence, the retina, which is already vulnerable due to relatively rapid ageing, can have an increased probability of being driven towards disease.

FH expression in the RPE is significant because the relatively mature RPE is pivotal in regulating tissue development on either side of it. Externally, the RPE guides periocular mesenchyme in choroidal formation^16,17^ and plays a role in development of scleral cartilage^18^. Internally, the apical RPE surface is the site of retinal mitosis and where a series of complex interactions take place between RPE cells and neuronal precursors orchestrating the pace of the cell cycle and the timing of cycle exit^19–21^. That FH may influence vascular development in the outer retina is supported by known interactions between it and vascular endothelial growth factor^22^ and by two key observations made in relatively young individuals with polymorphisms of complement. First, they have significant disruptions to choroidal blood flow^23^. Second, membrane attack complex deposition is present on their choroid vessels^24,25^. Both of these events occur decades before the potential development of AMD.

Complement is now thought to be important in CNS development^26^ and as FH is expressed in the RPE, which is so fundamental to ocular development, its absence here may be significant for retinal maturation and perhaps subsequently for AMD vulnerability. Here we test the hypothesis that CFH is critical for normal retinal development and that its absence may lay down a developmental footprint for increased probability of disease vulnerability much later in life.

## Results

**Figure 1:**
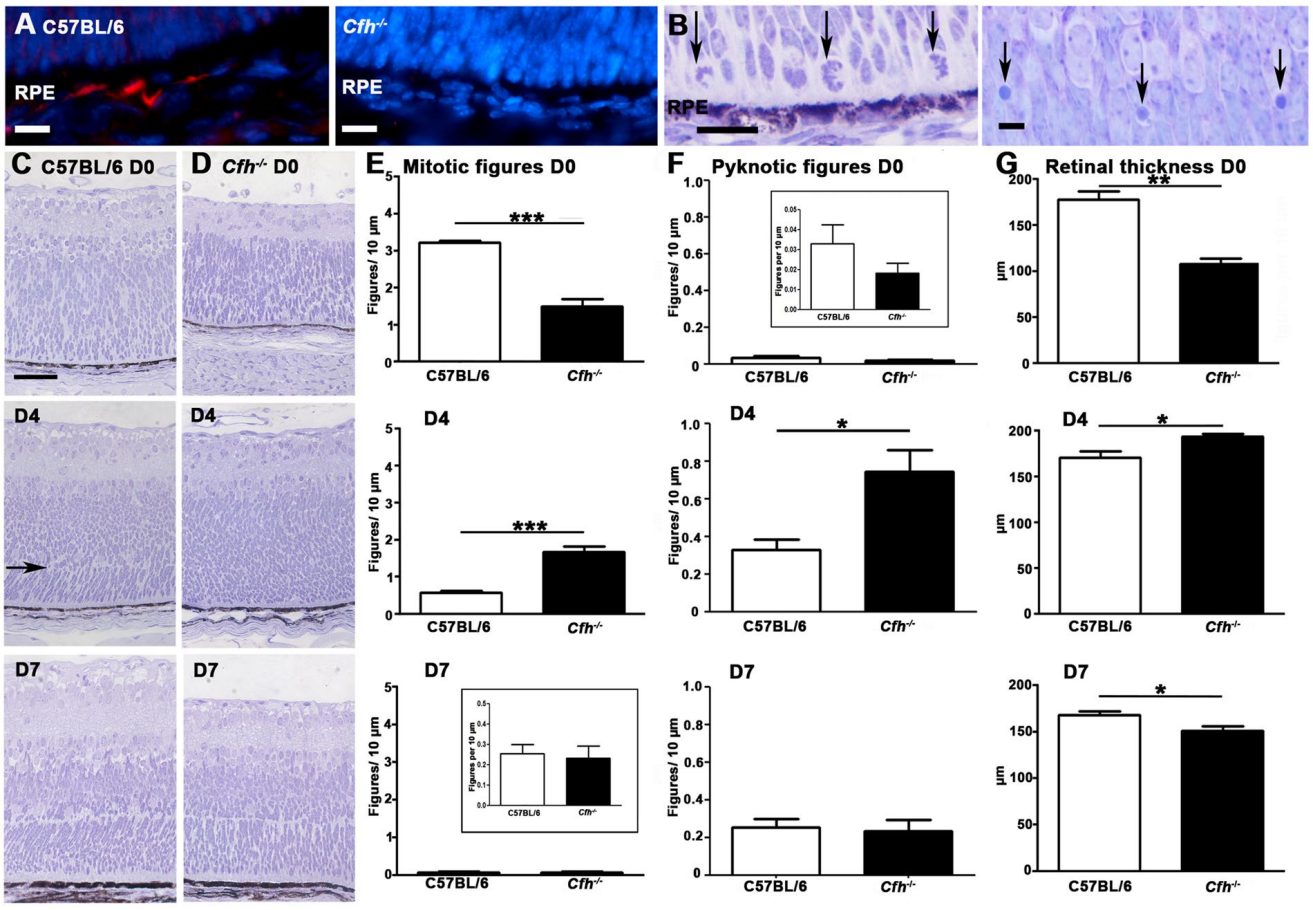
The course of retinal development in age matched *Cfh*^−/−^ and C57BL/6 mice. (**A**) FH was present in the RPE in C57BL/6 mice but absent in *Cfh*^−/−^ animals (n = 5 in each group). (**B**) Mitotic (left) and pyknotic (right) profiles were identified and counted in toludine blue stained plastic retinal sections in both C57BL/6 and *Cfh*^−/−^ (**C**,**D**) at D0 (n = 9 and 5 eyes), D4 (n = 5 and 5 eyes) and D7 (n = 5 and 5 eyes). (**E**) At D0 twice as many mitotic figures were found in C57BL/6 mice than *Cfh*^−/−^. This pattern reversed at D4 and by D7 when mitosis is almost over, numbers were similar. (**F**) At any stage fewer pyknotic profiles are found than mitotic figures. But again at D0 twice as many are found in C57BL/6 than *Cfh*^−/−^ and this pattern is reversed at D4 and reaches equivalence by D7. These data are consistent with a delay in retinal development in *Cfh*^−/−^ mice. This is reflected in the presence of an early outer plexiform layer in C57BL/6 at D4 (C arrow) that could not be found in *Cfh*^−/−^ mice. Also at this stage the neuropil in *Cfh*^−/−^ appeared qualitatively different and less organised. (**G**) Progressive changes in mitosis and pyknosis in the two genotypes were reflected in retinal thickness. Increased mitosis at D0 in C57BL/6 mice resulted in relatively thicker retinae, which reversed at D4 when mitosis caught up in *Cfh*^−/−^ prior to elevated cell death. By D7 retinae in the knock out mice were significantly thinner than in wild type. For statistical analysis, a Mann-Whitney U-test was used for comparison of two groups Error bars are standard error of the mean (SEM). Scale bars (**A**) Scale bar = 10 µm (**B**): Scale bar = 10 µm (**C**) Scale bar = 60 µm. Statistical significance: ^*^P ≤ 0.05: ^**^P ≤ 0.01 ^***^P ≤ 0.001.

In rodents, retinal mitosis peaks on or close to the day of birth gradually declining over the following 7 days. This overlaps with and is followed by a pronounced phase of cell death, with adult retinal architecture being established around day 7–10^19,20^. We stained wild type controls and *Cfh*^−/−^ mice for FH on D0, confirming its presence in RPE in C57BL/6 mice and its absence in *Cfh*^−/−^ mice (Fig. 1A).

Mitotic and pyknotic profiles (Fig. 1B) were counted at progressive stages along with measurements of retinal thickness in both mouse genotypes. Between D0 and D4, *Cfh*^−/−^ mice appeared to lag behind the C57BL/6 animals in terms of these features (Fig. 1C–F), but they converged towards the wild type pattern by D7. On D0 both the numbers of mitotic and pyknotic cells were greater in C57BL/6 mice. Measurements of retinal thickness (Fig. 1E–G) were also greater in the wildtype animals on D0 and D7 but not on D0 when mitosis peaked in the *Cfh*^−/−^ mice. In both genotypes mitotic figures were confined to the RPE surface while pyknotic profiles were widely distributed (Fig. 1B). By D4 mitosis declined in the C57BL/6 mice but was relatively elevated in the *Cfh*^−/−^animals. There was a relative increase in pyknosis in *Cfh*^−/−^ on D4 and the differences in retinal thickness between the two groups seen at D0 declined but were still significantly greater in *Cfh*^−/−^. By D7 there were no differences in mitosis or pyknosis between the groups, although the overall retinal thickness was significantly less in *Cfh*^−/−^. These data are consistent with delayed patterns of cell production and death in *Cfh*^−/−^ compared to C57BL/6.

**Figure 2:**
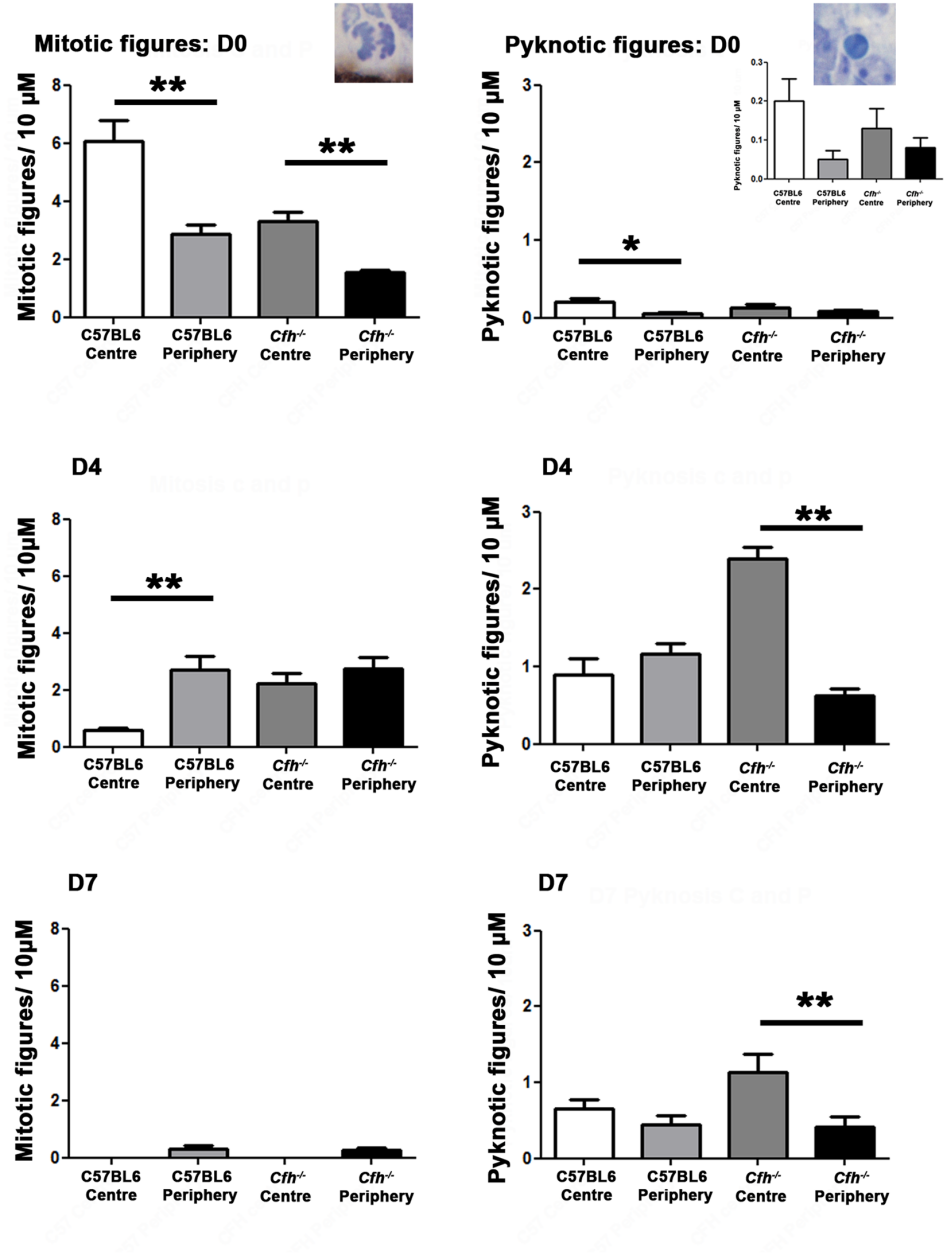
Spatial patterns of mitosis and pyknosis: centre versus periphery. Spatial patterns of mitosis and pyknosis were examined between the centre and periphery. At D0, as expected, there was a significant difference between mitosis in the centre and the periphery in the C57BL/6 animals (p = 0.004), which was also seen in the *Cfh*^−/−^ (p = 0.004). However, there are less mitotic figures in the C57BL/6 than the *Cfh*^−/−^. By D4, mitosis has reduced overall in the C57BL/6 and there were mitotic figures in the periphery than the centre. There was a trend towards this in the *Cfh*^−/−^. By D7, mitosis has entirely ceased in the centre in both groups with a small amount of residual mitosis in the periphery. Thus, for mitosis, both groups followed a centre to periphery pattern. For pyknosis, most pyknotic figures were found in the C57BL/6 group, in the centre with significantly less in the periphery (p = 0.006). *Cfh*^−/−^ also exhibited a centre to periphery gradient at D0 (but this was not significant 0.34). At D4, pyknosis appears to have already peaked in the C57BL/6, as the centre to periphery gradient was reduced. This was marked in the *Cfh*^−/−^, with much more pyknosis in the centre than the periphery (p = 0.004). At D7, pyknosis is reduced overall, with less pyknotic figures seen in the C57BL/6 group. However, a centre to periphery gradient exists in the Cfh^−/−^ group (p = 0.04), suggesting that pyknosis is not yet finished here. For statistical analysis, a Mann-Whitney U-test was used for comparison of two groups. Error bars are standard error of the mean (SEM). Statistical significance: ^*^P ≤ 0.05: ^**^P ≤ 0.01.

The vertebrate retina follows a centre to periphery pattern in its spatio-temporal development^19^. Hence, more mitotic figures are found centrally early in development and more in the periphery later. Centre to periphery patterns were clear in terms of mitosis and pyknosis in C57BL/6 mice at D0, D4 and D7. Although less marked, these patterns were also present in the *Cfh*^−/−^ mice. However, due to the delay in retinal development in these animals, pyknotic profiles were still dominant centrally at D7 (Fig. 2). These data are consistent with the notion that even though there are marked developmental deficits in *Cfh*^−/−^ mice, these do not appear to extend to a disruption of the centre to periphery gradient of retinal maturation.

**Figure 3:**
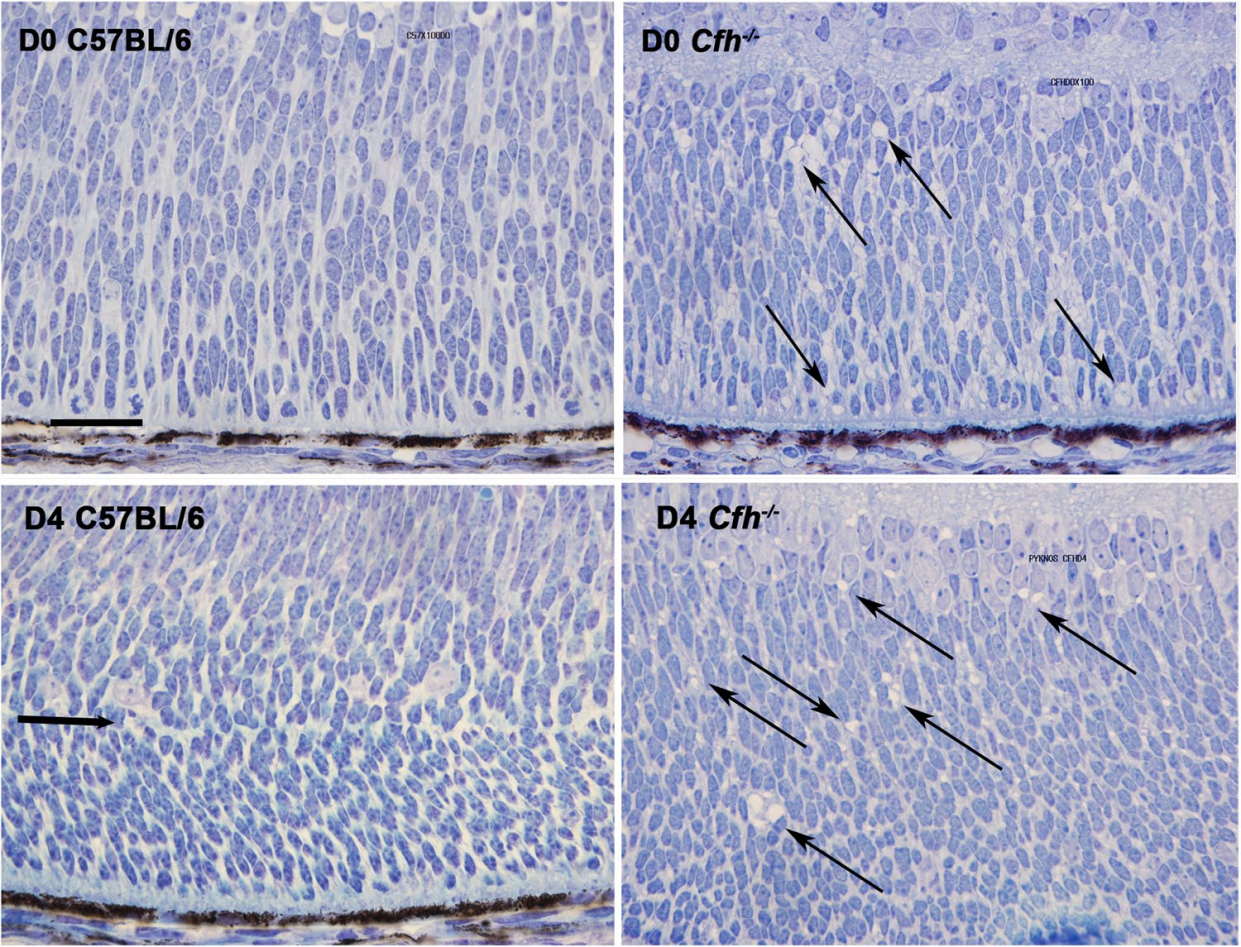
Representative images of central retinal regions in C57/BL6 and *Cfh*^−/−^ mice at D0 and D4. At these stages the *Cfh*^−/−^ mice displayed multiple extracellular vacuoles (arrows) within the neoblastic region of the neuropil that were not present in wild type animals. Few were identified at D7 (not shown). Scale bar = 50 µM.

**Figure 4:**
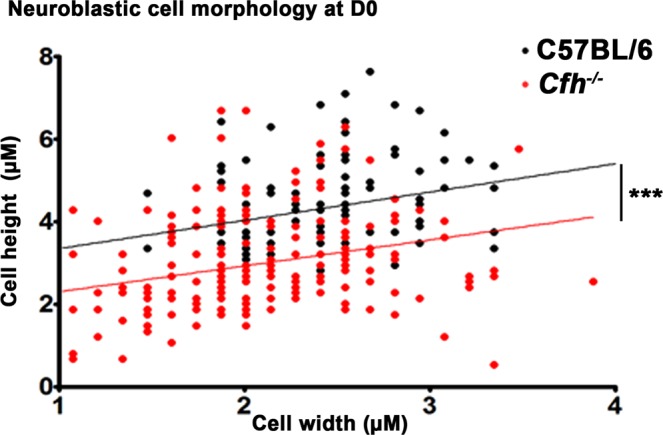
Cell size in neuroblastic region. Dimensions of neuroblastic cells were measured from photographs taken from 5 *Cfh*^−/−^ (N = 200) and 5 C57BL/6 mice *Cfh*^−/−^ and regression lines plotted for each. Overall, *Cfh*^−/−^ neuroblastic cells at D0 were smaller in both height and width, compared to C56/BL6. Many were also relatively flat. While the populations measured from the two genotypes overlapped they were significantly different. For statistical analysis, a Mann-Whitney U-test was used for comparison of two groups. Statistical significance ^***^P ≤ 0.001.

Along with these quantitative data other qualitative differences were apparent. The neuroblastic neuropil at D0 and D4 in *Cfh*^−/−^ animals consistently exhibited differences from that in the C57BL/6 mice. Cells were more congested in the *Cfh*^−/−^ compared to the wild type. When the neuropil was examined in detail in both genotypes at D0 and D4 multiple extracellular vacuoles were present within the neuroblastic regions in *Cfh*^−/−^ mice that were not present in the C57BL/6 animals (Fig. 3). These vacuoles were no longer present in the retina at D7 when mitosis was declining and retinal differentiation established. This feature was not seen in retinal regions that had differentiated in either genotype. Genotypic differences were not only confined to the extracellular environment but were also present within the cellular population within the neuroblastic area in the *Cfh*^−/−^ mice, as their morphology appeared differ from wild type. Hence, the long and short axes of these cells were measured in both genotypes at D0 and these data are shown in Fig. 4. Significant differences were found between cells in the *Cfh*^−/−^ mice and those in wild type. In *Cfh*^−/−^ neuroblastic cells were generally smaller in both width and height compared to those in the C57BL/6 mice. Hence, significant differences are present in both the morphology of the neuronal precursor population and their external environment at this key time period of development.

**Figure 5:**
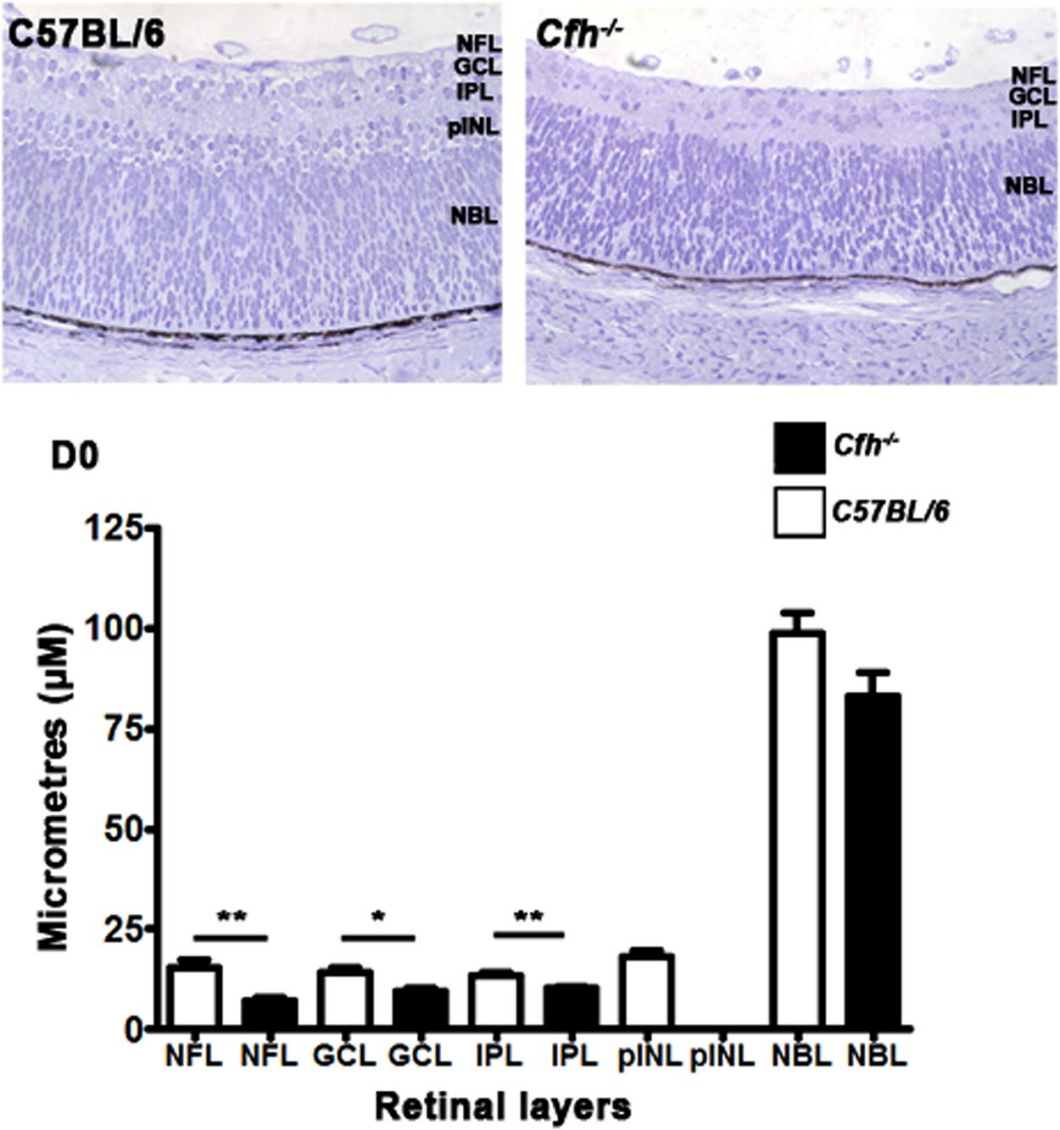
Patterns of retinal lamination on D0. Retinal lamination was examined at D0 by measuring the length of each retinal layer which was present. At D0, there were significant differences in the development of retinal layers. The nerve fibre layer (NFL), ganglion cell layer (GCL), inner plexiform layer (IPL) were all significantly greater in the C57BL/6 than the *Cfh*^−/−^. There was no prospective inner nuclear layer (pINL) in the *Cfh*^−/−^ but this was present in the C57BL/6. The neuroblastic layer (NBL) was thicker in the C57/BL6 but this was not significantly so. Thus, the *Cfh*^−/−^ lags behind the C57BL/6. Error bars are standard error of the mean (SEM). Mann Whitney U-test, one-sided. Scale bars Statistical significance: ^*^P ≤ 0.05; ^**^P ≤ 0.01.

There was also a concomitant significant delay in the formation of the laminar structure of the retina in the *Cfh*^−/−^ consistent with delayed patterns of cell production in these mice. This was first noticeable in the development of the presumptive outer plexiform layer. This region was present in C57BL/6 on D4 but remained largely absent from *Cfh*^−/−^ mice (Fig. 1C,D,D4 arrow). These data were confirmed when the thicknesses of the individual retinal layers were measured in the two genotypes. At D0 significant differences were present in the thickness of layers that could be identified, with those in *Cfh*^−/−^ being smaller (Fig. 5).

By D7 there were few obvious differences between the retinae in the two groups of mice other than that of overall retinal thickness (Fig. 1G). Differences in thickness were likely due to the elevated level of pyknosis seen on D4 in *Cfh*^−/−^ that reduced cellular populations by D7. At D7 the relative reduction seen in the *Cfh*^−/−^ retina was largely due to differences in the outer plexiform and ganglion cell layer. At 2 months of age differences in retinal thickness between the genotypes were no longer significant. Hence earlier abnormalities in *Cfh*^−/−^ mice do not leave an obvious signature on the overall histology seen after retinal differentiation.

**Figure 6:**
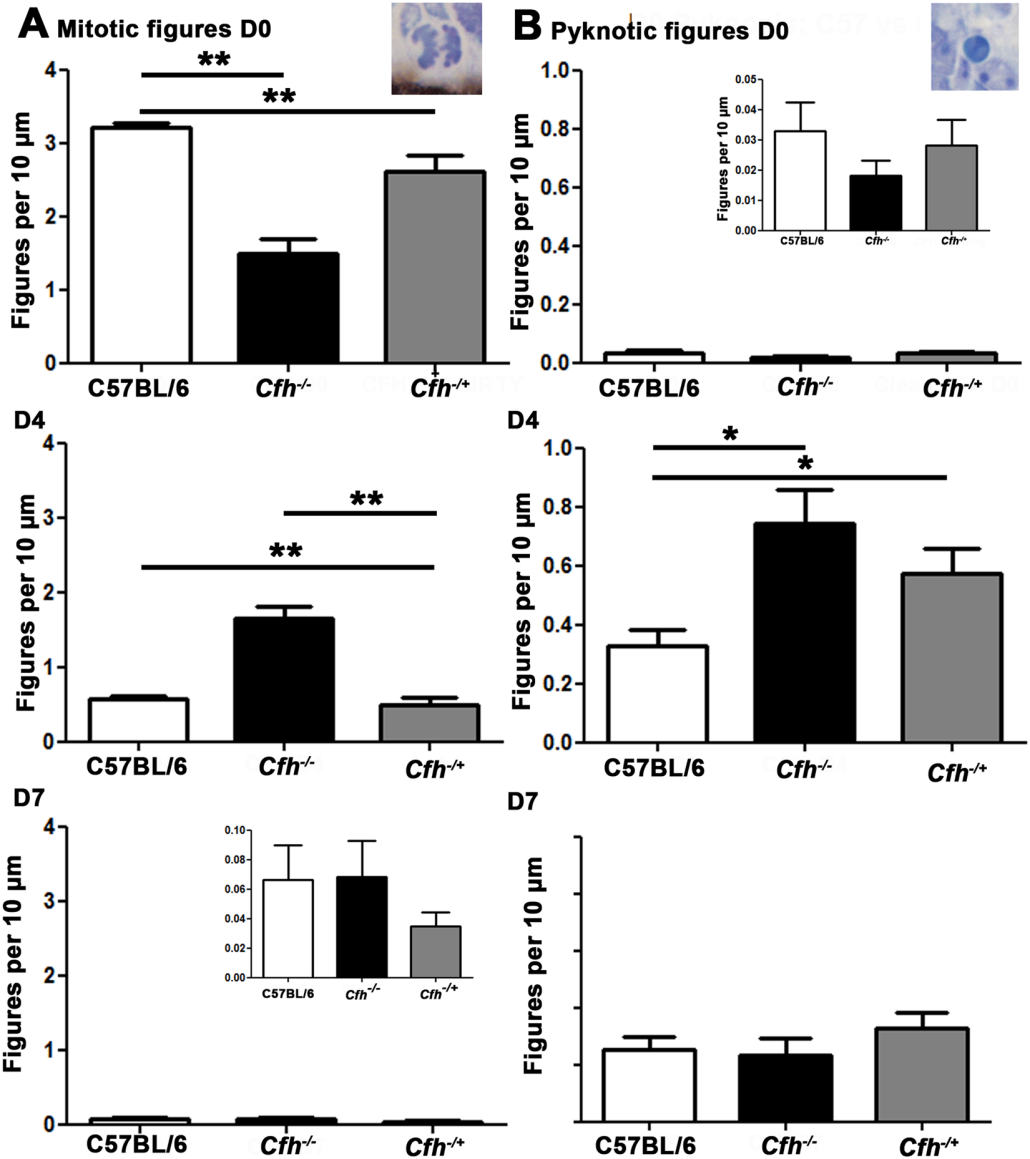
Mitotic figures D0 in *Cfh*^−/+^ compared to C57/BL6 and *Cfh*^−/−^. Mitotic and pyknotic figures were counted at the ventricular margin in *Cfh*^−/+^ animals to see the effect of a partial reduction in CFH. At D0, there was a small reduction in the number of mitotic figures difference between the C57/BL6 animals and the *Cfh*^−/+^ but this was not significant. There were no significant differences in pyknosis at D0 between the three groups. At D4, there was a significant difference between the groups (p = 0/006) in terms of retinal mitosis. There were a similar number of mitotic figures between the C57BL/6 and the *Cfh*^−/+^ but the *Cfh*^−/−^ was lagging behind. A statistically significant difference was found using a post-hoc between the *Cfh*^−/−^ and C57/BL6 but not between C57/BL6 and *Cfh*^−/−^. This was significant using a one-tailed Mann Whitney U-test (p = 0.004). With pyknosis, the *Cfh*^−/+^ had a level of pyknosis between the C57BL/6 and the *Cfh*^−/−^. Overall, there was a significant difference between the two groups (p = 0.034) and there was a significant difference between C57BL/6 and *Cfh*^−/−^ but not between the C57BL/6 and *Cfh*^−/+^. At D7 there were no differences between the groups for both mitosis and pyknosis. These results suggest that a partial reduction in FH and the absence of FH have different effects on retinal development. For statistical analysis, a Mann-Whitney U-test was used for comparison of two groups and a Kruskal-Wallis was used for comparison of three-groups with Dunns multiple comparison test as a post-hoc test.

Here our comparisons are between wild type and *Cfh*^−/−^, however, in AMD the association with CFH is that of polymorphisms, not a deletion. Hence, a *Cfh*^−/+^ model may be more relevant. Consequently, we repeat the analysis of patterns of mitosis and pyknosis to include this genotype. Data from these mice show significant deficits in mitosis and pyknosis largely similar to those found in the knock out animals, but of a reduced magnitude. Hence, deficits are resent in *Cfh*^−/+^ but to a lesser extent than found in *Cfh*^−/−^ animals consistent with there being a partial reduction in CFH expression impacting on retinal development (Fig. 6).

**Figure 7:**
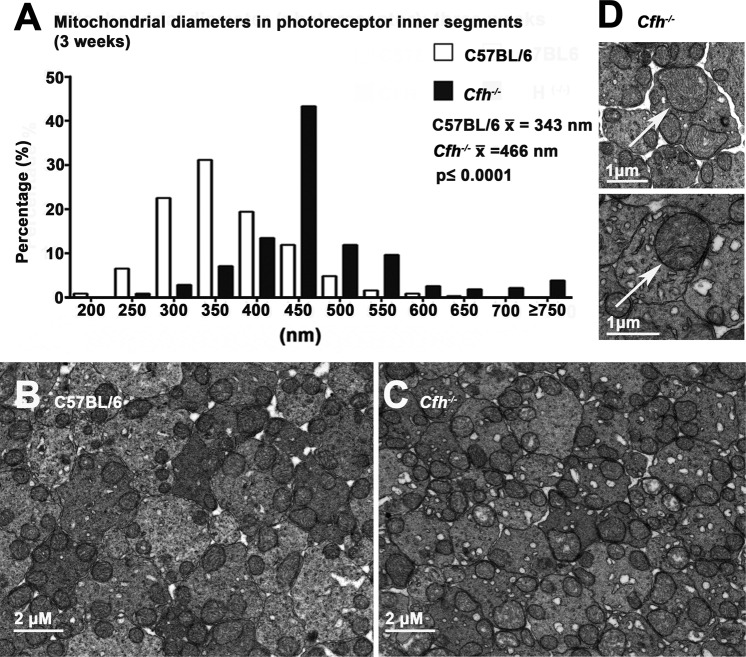
Mitochondrial size in photoreceptor inner segments at 3 weeks. Sections were cut across the inner segment short axis in the two genotypes (C57/ BL6 n = 3; *Cfh*^−/−^ n = 2 animals). (**A**) When mitochondrial diameters were measured they were significantly larger *Cfh*^−/−^ than C57BL/6 by approximately 30%. (**B**,**C**) Show examples in each genotype and in (**D**) larger power images focus on two examples of enlarged mitochondria (arrow) in the knock-out mice. Enlarged mitochondria are indicative of senescence. For statistical analysis, a Mann-Whitney U-test was used for comparison of two groups. Statistical significance. p ≤ 0.001.

ATP production declines prematurely in the *Cfh*^−/−^ retina long before an aged retinal phenotype is established^27^, implying that that there may be mitochondrial disruption in this model. Mitochondria in the outer retina were examined at the electron microscope level on D21, which is when the retina has fully differentiated and the patterns of outer retinal integration are established^19^. We examined mitochondria in the outer retina in photoreceptors and in the RPE, which is the site of disease development. In normal mice mitochondria in photoreceptor inner segments are densely packed and aligned along the cell membrane close to the cells oxygen supply^28^. Here, Mitochondria in inner segments were viewed across both the inner segment long axis and across the transverse axis. In transverse section many mitochondria in the *Cfh*^−/−^ mice were enlarged, which is associated with ageing and senescence. In the analysis, only clearly circular profiles were measured, however, when the distribution of mitochondrial diameters were compared between the *Cfh*^−/−^ and C57BL/6 mice it was clear that while in both genotypes distributions followed a similar overall skewed distribution, that of the *Cfh*^−/−^ was shifted significantly to the right (Fig. 7). In C57BL/6 mice the mean mitochondrial diameters peaked at around 300–350 nm, while in the *Cfh*^−/−^ mice the peak was located at 400–450 nm, which is an increase of around 30% and statistically significant (p < 0.001).

**Figure 8:**
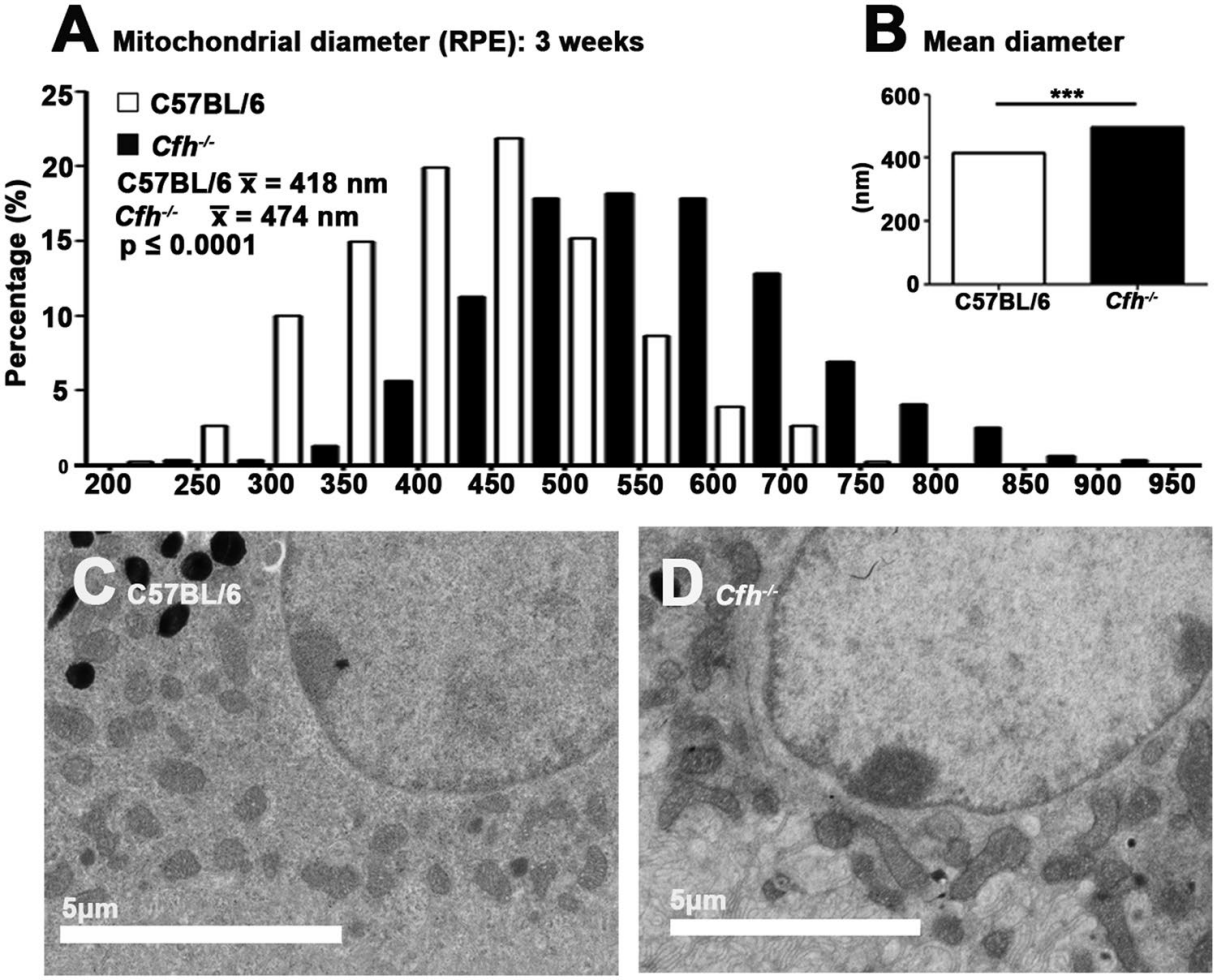
Mitochondrial size in the RPE at 3 weeks in RPE. Mitochondria are shown in both genotypes (C57/ BL6 n = 3; *Cfh*^−/−^ n = 2 animals). As with data from inner segments at 3 weeks, mitochondria in the *Cfh*^−/−^ were significantly larger than in the wild type by around 20% (**A**,**B**) Statistical significance: ^*^p ≤ 0.05 (**C**,**D**) show representative images of mitochondria in the two genotypes. For statistical analysis, a Mann-Whitney U-test was used for comparison of two groups. Statistical significance: ^***^p ≤ 0.0001, where n is the number of mitochondria (<100 in each group).

In the RPE there is no marked alignment of mitochondria with the plasma membrane in wild type mice. However, when the diameters of mitochondria were examined in the RPE in both genotypes a similar pattern was found to that in photoreceptor inner segments, with mitochondria in the *Cfh*^−/−^ being enlarged. Another feature of RPE mitochondria seen in the *Cfh*^−/−^ mice appeared to be differences in their morphology (Fig. 8) that may have been due to different patterns of fusion and fission. However, in both the photoreceptors and the RPE their internal structure of mitochondrial appeared relatively normal in the *Cfh*^−/−^.

**Figure 9:**
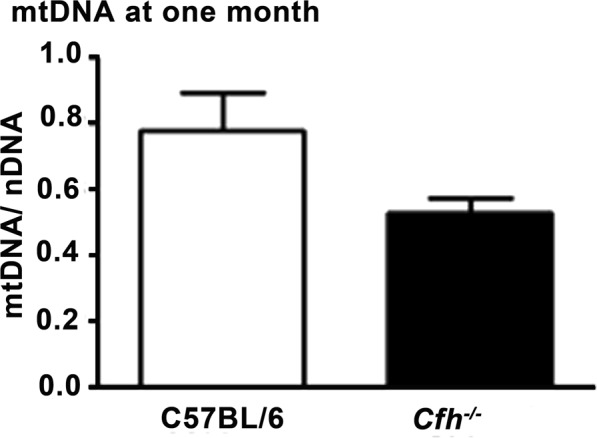
mtDNA content. Total mitochondrial DNA (mtDNA) measured at one month from the retinae of both genotypes. This was reduced by approximately 30% in *Cfh*^−/−^ animals compared to that found in C57BL/6, consistent with there being fewer mitochondria in the knock out. However, this difference did not reach statistical significance (p = 0.055). C57BL/6 n = 4; *Cfh*^−/−^ n = 5 animals. Mann-Whitney U-test was used for comparison of two groups. Error bars are standard error of the mean (SEM).

The EM evidence reveals that once the mature architecture of the retina is established in young *Cfh*^*−/−*^ mice there are significant changes in their mitochondria that are consistent with patterns seen in degeneration/senescence. If correct, then this may be reflected by changes in total mtDNA. This was measured in both groups and found to be reduced in the *Cfh*^−/−^ mice by 30% compared to C57BL/6 controls, although this just failed to reach statistical significance (p = 0.055). Hence, not only are mitochondria abnormal in the young *Cfh*^−/−^ there are likely to be less of them compared to those found in wild type animals (Fig. 9).

**Figure 10:**
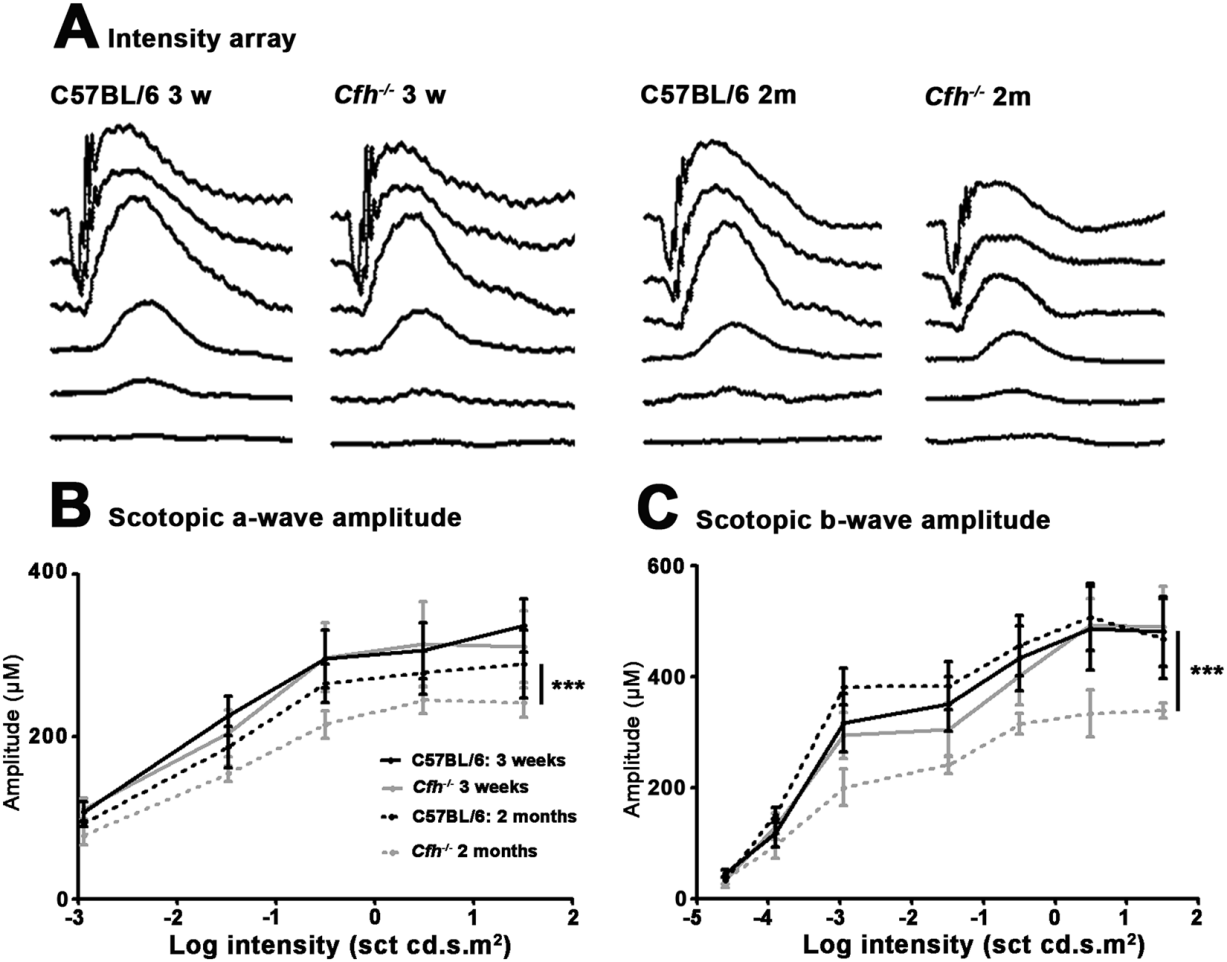
Electroretinograms (ERGs). These were recorded for progressive light intensities series from *Cfh*^−/−^ and C57BL/6 mice at D21 and D56 to scotopic stimuli that resulted in responses predominantly from the rod photoreceptor population. (**A**) Representative ERGs showing scotopic a-waves (photoreceptor function) and b-waves (post-receptoral function) in the C57BL/6 and *Cfh*^−/−^. At 21 days the negative a-wave and subsequent positive b-wave are similar between the genotypes. However, by D56 both wave forms are reduced in the *Cfh*^−/−^ mice by approximately 30%, consistent with reduced retinal function. Peak waveforms were measured for the a-wave (**B**) and the b-wave (**C**) between animals in both groups. a 2-way ANOVA test was used for comparison of two or more groups. In both cases significant reductions were consistently found in *Cfh*^−/−^ mice at D56. There were no obvious differences in the timing of the waves between the genotypes. ^***^P < 0.001. n = 5 animals in all groups. Error bars are standard error of the mean (SEM).

To match degenerative changes in mitochondria to any potential decline in retinal function, ERGs were recorded on *Cfh*^−/−^ and C57BL/6 mice at D21 and D56. These were to both rod (scotopic) and cone (photopic) function and to both the photoreceptor generated a-wave and the post-receptoral b-wave. No differences were found in the cone driven photopic function, but this is generated by only 3% of the photoreceptor population^29^. There were no significant differences in scotopic function between the two genotypes at D21. However, on D56 in *Cfh*^−/−^ mice there was a significant decline in the response amplitudes of both the scotopic a-wave and b-wave at higher intensities of rod function. The significant reduction in the a-wave was approximately 15%, while at higher intensities the significant reduction in the b-wave was approximately 30%. There were no differences in their timing (Fig. 10).

## Discussion

These results show that in *Cfh*^−/−^ mice there are significant disruptions to the development of the retina in terms of the temporal patterns of cell production and cell death and cellular morphology. Significant abnormalities are also present in both the developing extra-cellular matrix and in the morphology of cells that are within the neuroblastic region between D0 and D4–7. While the overall architecture of the *Cfh*^−/−^ retina achieves a relatively normal configuration at the end of development, its thickness remains significantly reduced at D7 when retinal differentiation has largely been achieved. Given the significant disruption to the timing of these key events, it is likely that the *Cfh*^−/−^ retina contains flaws in its adult form that may predispose it to later degeneration, particularly when mice are exposed to a normal pathogen loaded open environment^30^. Our data also imply that developmental deficits are present in *Cfh*^−/+^ mice that would be relevant to studies that have demonstrated deficits in humans with polymorphisms of complement decades before development of AMD.

*Cfh*^−/−^ mice do not develop a retinal phenotype in a SPF environment^30^. All of our mice were maintained in an open environment. However, limited observations on patterns of mitosis and cell death made in directly related *Cfh*^−/−^ mice maintained in a SPF environment failed to show significant differences from the mice maintained in an open environment. This supports the notion that our results reflect a fundamental developmental feature unrelated to environmental influence.

The RPE is known to play a major role in regulating retinal mitosis. When ablated during development the retina is lost and the eye becomes microphthalmic^31^. When pigment is absent from the RPE, mitosis is deregulated and delayed and many cells fail to leave the cycle and die, giving rise to the mature deficits seen in albinism^32^. The results reported here partially mirror those found in albinism with similar delayed and disrupted maturation, although in the case of albinism such deficits are the result of the absence of DOPA, an early upstream melanin precursor in the RPE that regulates the pace of the cell cycle^20,33^.

It is unclear how the developmental deficits we describe become associated with such marked mitochondrial decline. For simplicity, our study has confined its analysis of mitochondria to the differentiated retina, although it is possible that mitochondrial abnormities in *Cfh*^−/−^ mice are present earlier. However, investigation of earlier mitochondria events become clouded while mitosis is active as this involves their segregation during cell division. Likewise they play a major role in initiating the signaling mechanisms that result in apoptosis. There are multiple points of interaction between elements in the innate immune system and mitochondria at maturity, and West *et al*.^34^ have argued that mitochondria are centrally positioned hubs in multiple signaling pathways in the innate immune system. There are many bridge points between mitochondria and induction of inflammatory and degenerate processes both in specific tissues^35^ and also in the circulation^36^. But the mechanism here in the retina is less clear as is the issue of exactly what is triggering the cascade of changes we describe.

A clearer picture is evolving of the role of complement in development^37,38^. Here a key observation is that complement plays a role in the CNS in pruning of excess connections that are a feature of CNS maturation^39,40^. This includes regulating apoptosis where mitochondria are known players. A mechanism for removal of excess neuronal branching is the deposition of C3 on individual branches which flags them for pruning. In the absence of FH, C3 will not be regulated appropriately and may be elevated^37^. These abnormal developmental factors appear to establish vulnerability in the *Cfh*^−/−^ retina that is exploited by age.

The nature of the interactions between FH in the RPE and mitotic retinal precursors remains unclear and beyond the limitations of this study. However, they are likely key to potentially unravelling the developmental footprint for AMD that we have revealed. In the developing albino retina that shares so many parallels with the *Cfh*^−/−^ mouse, abnormities are not confined to mitosis. They are also present in the orientation of mitotic profiles and patterns of gap junctional connectivity between RPE and dividing cells^21,33^. But the RPE/neuroblastic interface is a highly active region that changes on a day by day basis through development as cells come out of the cell cycle and the cycle itself progressively lengthens^19^. If there are fundamental differences in these features between wild type and *Cfh*^−/−^ mice as supported by our data, it is likely that the fine tuning of retinal development in *Cfh*^−/−^mice  and its patterns of internal connectivity are different from the wild type. It is also likely that there are shifts in the sizes of different neuronal population that are generated in different time periods^41^. To date, we have no data on these potentially important differences.

Given the mitochondrial abnormality established in the *Cfh*^−/−^ mice and the subsequent decline in ATP production^27^ it is not surprising that there was a reduction in the magnitude of the ERG. Measuring ATP and recording ERG responses are relatively crude metrics and probably fail to reveal small changes that may be significant for the organism. Hence, it is possible that given the abnormalities in mitochondria found at 3 weeks there were disruptions to retinal function at this earlier stage that our methods could not reveal.

The reduction in the ERG shown in *Cfh*^−/−^ mice was confined to the rod response and to its amplitude not its timing. In this respect it may be significant that mitosis was markedly reduced in *Cfh*^−/−^ at the peak of rod production that is around D0 and was followed later by elevated pyknosis. Reduced rod number would likely be reflected in the magnitude of the ERG but not its timing.

Our results reveal for the first time that FH is a key player in sculpting retinal development. Further, in its absence there are fundamental deficits in mouse retinal development that likely impact on the ability of the retina to cope with ageing, as the *Cfh*^−/−^ mouse suffers marked retinal pathology and partial collapse of the choroid^42,43^. These data along with those derived from humans with polymorphisms of FH argue^23^ that there may be a significant developmental foot print for AMD that has not previously been appreciated and may change the way this disease is viewed.

## Methods

### Animals

All animals were used with University College London ethics committee approval and conformed to the United Kingdom Animal License (Scientific Procedures) Act 1986 (UK). UK Home Office project license (PPL 70/6571). They were fed on standard diet and maintained on a 12/12 h light cycle. They were maintained in an open environment and not kept under specific pathogen free (SPF) conditions^30^. *Cfh*^−/−^ were on a C57BL/6 J background and back-crossed 10X and regularly genotyped. Animal numbers for each part of the study were as follows. For immunohistochemistry to identify FH on the day of birth, 10 mice were used**:** C57BL6 (n = 5), *Cfh*^−/−^ (n = 5). For resin embedded histology in the analysis of mitotic and pyknotic profiles, 6 cohorts of C57/BL6 and *Cfh*^−/−^ mice were used aged D0 (n = 9 and 5 eyes), D4 (n = 5 and 5 eyes) and D7 (n = 5 and 5 eyes). *Cfh*^−/+^ were also used for comparison in resin at D0 (n = 3 and 3 eyes), D4 (n = 5 and 5 eyes) and D7 (n = 5 and 5 eyes). At 2 months, two cohorts of mice were used (C57BL6 n = 5; *Cfh*^−/−^ n = 5 eyes).

For ERGs, four cohorts of mice were used aged 3 weeks (*Cfh*^−/−^ n = 5; C57/BL6 n = 5 animals) and 2 months (C57/BL6 n = 5; *Cfh*^−/−^ n = 5 animals). For mtDNA extraction, two cohorts of mice were used aged 1 month (C57BL/6 n = 4; *Cfh*^−/−^ n = 5 animals). For EM, two cohorts of mice were used at three weeks (C57/BL6 n = 3; *Cfh*^−/−^ n = 2 animals). Three weeks was selected because the retina had by this time adopted its adult configuration. More than 250 mitochondria were measured per eye.

### Resin embedded histology

Mice were culled by cervical dislocation and their eyes removed and placed in 2% paraformaldehyde and 2% glutaraldehyde in phosphate buffered saline for 24 hours. The tissues were then washed in PBS and dehydrated through a graded series of ethanols before being infiltrated, polymerised and embedded in Technovit 7100 historesin solution (Taab Laboratories equipment, UK). Resin sections were cut near the optic nerve head at 5 μm mounted on glass slides and stained with 1% Toludine Blue and mounted in Depex mounting medium and coverslipped.

Mitotic and pyknotic figures were counted in four closely spaced central sections in each eye, with numbers normalized for the length of section. The thickness of the developing retinal layers were measured using the grid of the microscope and calibrating it with a graticule. Images were captured using a 40X objective lens and 10X eyepieces in JPEG format using a bright-field microscope with a 24-bit colour images at 3840 × 3072 pixel resolution using a Nikon DXM1200 digital camera. For statistical analysis, a Mann-Whitney U-test was used for comparison of two groups and a Kruskal-Wallis was used for comparison of three-groups with Dunns multiple comparison test as a post-hoc test. Data were analysed using GraphPad Prism version 5.0 for windows (GraphPad, San Diego, USA).

### Immunohistochemistry

For identification of the presence of FH, mice were culled by cervical dislocation and their eyes were removed and fixed in 4% paraformaldehyde in phosphate buffered saline (PBS), pH 7.4, for 1 hour, cryopreserved in 30% sucrose in PBS and embedded in optimum cutting temperature (OCT) compound (Agar Scientific Ltd). Cryostat sections were cut at 10 µm and thaw-mounted onto charged slides. These were air dried and then washed with 0.1 M PBS. Sections were incubated for 1 h in a 5% Normal Donkey serum in 0.3% Triton X-100 in PBS, pH 7.4. Thereafter, they were incubated overnight at room temperature with a goat polyclonal FH (1:1000, Covance, Princeton, USA) which was diluted in 1% Normal Donkey Serum in 0.3% Triton X-100 in PBS. Negative controls had the primary antibody omitted. The sections were subsequently X3 in 0.1 M PBS at 5 minute intervals, then incubated for 1 h in a donkey-anti-goat secondary antibody conjugated with Alexa Fluor 568 (Invitrogen) diluted in 2% Normal Donkey Serum in 0.3% Triton X-100 in PBS at a dilution of 1:2000. The sections were washed again X3 with 0.1 M PBS at 5 minute intervals. Nuclei were subsequently stained with 4′, 6-diamidino-2-phenylindole (Sigma, UK) for 1 min. Slides were rewashed as above then washed X4 in Tris buffered Saline (pH 7.5). The slides were mounted in Vectashield (VECTOR Laboratories), coverslipped and sealed.

### Electroretinogrames

The ERG was undertaken under scotopic (intensity sequence) conditions with mice dark adapted overnight in a light-proof ventilated box. Mice were anaesthetised with 6% Ketamine, (National Veterinary Services Ltd, UK) 10% Dormitor (National Veterinary Services Ltd, UK) and 84% sterile water at 5 ul/g intraperitoneal injection. Their pupils were dilated (1% Tropicamide, MINIMS, Bausch and Lomb, France). The cornea was lubricated with Viscotears (Novartis, Switzerland). Ground and reference subdermal electrodes were placed subcutaneously near the hindquarter and between the eyes and the mouse placed on a heated pad (37 °C). Recording gold electrodes (C.H. Electronics) were placed on and measured from the cornea of the right eye. The ERG equipment included a full-field ganzfeld stimulator covering the mouse body (Espion, Diagnosys LTD, Cambridge). Scotopic ERGs were undertaken with increasing stimulus strengths using a 6500 K white light at 1.25 × 10^−6^, 1.1 × 10^−5^, 3.3 × 10^−4^, 0.03, 0.3, 3.1 and 31.9 cd s/m^2^. At the lower 4 intensities, 30 readings were taken. At the higher 3 intensities, 3 recordings were taken. ERG data was obtained using proprietary Espion software from Diagnosys (Cambridge, UK). For statistical analysis, a 2-way ANOVA test was used for comparison of two or more groups. Data were analysed using GraphPad Prism version 5.0 for windows (GraphPad, San Diego, USA).

### mtDNA

Mice were culled by cervical dislocation. Eyes were collected and the retina removed and homogenised in 100 µl of chilled Homogenisation medium (0.32 M Sucrose, 1 mM EDTA, 10 mM Tris-HCL, pH 7.4). This was centrifuged at 1000 g for 5 min at 4 °C. The supernatant was further centrifuged at 15,000 g for 2 min at 4 °C with the resultant pellet re-suspended in homogenising medium and spun down a final time at 15,000 g for 2 min at 4 °C. The final pellet was equilibrated in 50 µl of Incubation buffer (25 mM Sucrose, 100 mM KCl, 0.05 mM EDTA, 5 mM MgCl_2_, 10 mM Tris-HCl, 10 mM H_3_PO_4_, pH 7.4). The acquired fractions were processed for DNA quantification by incubating in DNA Digestion Buffer containing Proteinase K (50 mM Tris-HCl, 100 mM EDTA, 100 mM NaCl, 1% SDS, pH 8.0) and then extracted with Phenol/Chloroform/Iso-amyl alcohol (PCI). The fractions were then spun down at top speed for 5 min at 4 °C, the upper phase was further spun down twice at top speed for 5 min at 4 °C. The final pellet was stored in 50 µl of TE Buffer (10 mM Tris-HCl, 1 mM EDTA, pH 8.0). DNA concentration was determined spectrophotometrically (NanoDrop ND-1000). For statistical analysis, a Mann-Whitney U-test was used for comparison of two groups. Data were analysed using GraphPad Prism version 5.0 for windows (GraphPad, San Diego, USA).

### Electron microscopy

Eyes were fixed for a minimum of 2 h in Karnovsky’s EM fixative (2.5% Glutaraldehyde, 2% paraformaldehyde, 0.08 M sodium cacodylate). The anterior portion of the eye including the lens was removed. Samples were washed X 2 in 0.1 M cacodylate buffer and were fixed for 1 h in osmium tetroxide (2% in water). They were then washed X 3 in distilled water and dehydrated in a series of alcohols and transferred to Propylene oxide (2 × 30 min) before being infiltrated with 50% araldite resin/50% propylene oxide overnight. Samples were infiltrated with 100% araldite resin for 8 h and heated at 60 °C to cure. Ultathin 70 nm sections were cut with a diamond knife on a Leica Ultracut S, stained with Reynold’s lead citrate and imaged with and a Jeol 1010 TEM fitted with a Gatan digital camera. For statistical analysis, a Mann-Whitney U-test was used for comparison of two groups. Data were analysed using GraphPad Prism version 5.0 for windows (GraphPad, San Diego, USA). n = the number of mitochondria (>100 in each group).
